# Real-Time Driver Attention Detection in Complex Driving Environments via Binocular Depth Compensation and Multi-Source Temporal Bidirectional Long Short-Term Memory Network

**DOI:** 10.3390/s25175548

**Published:** 2025-09-05

**Authors:** Shuhui Zhou, Wei Zhang, Yulong Liu, Xiaonian Chen, Huajie Liu

**Affiliations:** 1 CGNPC Uranium Resources Co., Ltd., Beijing 100084, China; 2 Suzhou Automotive Research Institute (Wujiang), Tsinghua University, Suzhou 215200, China

**Keywords:** head pose estimation, convolutional neural network, long short-term memory, binocular vision

## Abstract

Driver distraction is a key factor contributing to traffic accidents. However, in existing computer vision-based methods for driver attention state recognition, monocular camera-based approaches often suffer from low accuracy, while multi-sensor data fusion techniques are compromised by poor real-time performance. To address these limitations, this paper proposes a Real-time Driver Attention State Recognition method (RT-DASR). RT-DASR comprises two core components: Binocular Vision Depth-Compensated Head Pose Estimation (BV-DHPE) and Multi-source Temporal Bidirectional Long Short-Term Memory (MSTBi-LSTM). BV-DHPE employs binocular cameras and YOLO11n (You Only Look Once) Pose to locate facial landmarks, calculating spatial distances via binocular disparity to compensate for monocular depth deficiency for accurate pose estimation. MSTBi-LSTM utilizes a lightweight Bidirectional Long Short-Term Memory (Bi-LSTM) network to fuse head pose angles, real-time vehicle speed, and gaze region semantics, bidirectionally extracting temporal features for continuous attention state discrimination. Evaluated under challenging conditions (e.g., illumination changes, occlusion), BV-DHPE achieved 44.7% reduction in head pose Mean Absolute Error (MAE) compared to monocular vision methods. RT-DASR achieved 90.4% attention recognition accuracy with 21.5 ms average latency when deployed on NVIDIA Jetson Orin. Real-world driving scenario tests confirm that the proposed method provides a high-precision, low-latency attention state recognition solution for enhancing the safety of mining vehicle drivers. RT-DASR can be integrated into advanced driver assistance systems to enable proactive accident prevention.

## 1. Introduction

Road safety constitutes a core concern in the development of intelligent transportation systems. Driver distraction represents one of the critical human factors contributing to traffic accidents [1,2]. The total monetized societal impact of motor vehicle crashes in the United States amounted to USD 83.6 billion in 2010, with approximately 15% attributable to distracted driving [3,4]. Consequently, developing efficient and reliable methods for detecting driver attention states holds significant theoretical value and practical importance for enhancing driving safety and reducing accidents. Current driver attention state recognition methods are primarily classified into three categories: physiological signal-based [5,6,7], driving behavior-based [8], and computer vision-based analysis [9]. Physiological signal-based methods (e.g., monitoring EEG, ECG, GSR) can directly reflect the driver’s physiological state [10,11,12]. But they typically rely on contact devices, suffering from limitations such as high cost, poor comfort, and potential interference with driving operations [10]. Driving behavior-based methods infer attention states indirectly by analyzing steering wheel operations, braking patterns, etc., offering non-intrusive advantages [13,14,15]. Nevertheless, their detection efficacy is susceptible to complex traffic environments, road conditions, and individual driving habit variations, often exhibiting insufficient generalization capability and real-time performance. In contrast, computer vision-based methods, capturing and analyzing visual features like facial expressions and head pose, combine non-intrusiveness, low deployment cost, and strong scalability, emerging as the dominant research and application direction [16,17].

Among visual approaches, facial expression analysis is vulnerable to illumination variations and occlusion [18,19]. Head pose estimation, inferring the driver’s focus of attention by analyzing spatial position and orientation changes of the head, is considered a preferred solution for large-scale in-vehicle deployment due to its relative simplicity, moderate computational demands, good real-time performance, strong non-intrusiveness, and controllable cost. Within computer vision-based head pose estimation, traditional methods include 2D image analysis techniques based on feature point detection or template matching [20]. But their accuracy is limited by the lack of depth information in monocular images, resulting in suboptimal performance. Three-dimensional methods based on geometric models (e.g., 3D facial modeling, Direct Linear Transformation) or point cloud alignment improve accuracy but incur high computational complexity, hindering real-time pose estimation [21]. The rise of deep learning has significantly advanced this field. Convolutional Neural Networks (CNNs) learn effective features end-to-end from raw images [22,23,24]. Transformer networks capture long-range dependencies via self-attention [25]. Hybrid architectures (e.g., CNN-Transformer) attempt to fuse their strengths for enhanced feature representation [26]. Nevertheless, existing methods still face significant challenges in robustness under complex driving scenarios (e.g., severe illumination changes, occlusion, rapid motion) and computational efficiency for millisecond-level real-time responsiveness.

Furthermore, unimodal approaches exhibit inherent limitations [27]. Multimodal fusion strategies, integrating visual, physiological, and behavioral information, theoretically enhance detection accuracy and robustness [28]. Current fusion methods explore advanced techniques such as attention mechanisms for dynamic weighting and federated learning for privacy protection [29]. Despite advances, multimodal fusion encounters practical deployment barriers, including data synchronization difficulties, drastically increased system complexity, and substantial computational resource demands [18]. In summary, vision-based head-pose estimation holds considerable promise for driver-attention monitoring. Nevertheless, monocular approaches suffer from limited accuracy owing to the absence of explicit depth cues, whereas point-cloud-based and multi-sensor fusion methods are still constrained in real-time performance. Moreover, the overwhelming majority of existing investigations have not been tailored to the specificities of mining-truck drivers. Consequently, a rapid yet accurate technique for assessing the attentional state of mining-truck drivers remains an open challenge.

To address the aforementioned challenges, this paper proposes RT-DASR. Within this framework, the BV-DHPE module is dedicated to estimating head orientation. BV-DHPE leverages a calibrated stereo rig to simultaneously capture facial imagery and dense depth, thereby eliminating the depth-ambiguity inherent in monocular setups. An off-the-shelf YOLO11n Pose [30] model is adopted to directly regress a sparse set of facial landmarks. This single-shot paradigm circumvents the conventional two-stage pipeline of face detection followed by landmark localization, yielding both higher precision and lower latency. Facial landmarks and facial depth information are used to estimate the angle of the head posture. The extracted head-pose sequence, together with the driver’s dynamic gaze region and the vehicle velocity, is then forwarded to the MSTBi-LSTM module. By bidirectionally fusing these three low-dimensional yet temporally aligned cues, MSTBi-LSTM continuously discriminates subtle transitions in driver attention without incurring the computational burden typical of high-dimensional multi-modal fusion. Owing to the real-time capability of YOLO11n-Pose and the lightweight architecture of the Bi-LSTM network, RT-DASR achieves accurate, real-time recognition of mining-truck driver attention states under stringent latency constraints.

The main contributions of this work are as follows:

(1) Binocular Vision Depth-Compensated Head Pose Estimation component

Addressing the inaccuracy in pose estimation caused by depth deficiency in monocular images, we adopt a binocular camera framework. Key facial landmarks (inner/outer eye corners, eyebrow outer corners, nostrils) are extracted using the YOLO11n Pose model. Spatial distances are computed via binocular disparity, enabling precise head pose angle estimation. Experimental validation demonstrates a 44.7% reduction in MAE compared to monocular approaches.

(2) Multi-source Temporal Bidirectional Long Short-Term Memory Feature Fusion component

This method bidirectionally fuses temporal sequences of head pose dynamics, real-time gaze region shifts, and vehicle speed information. It utilizes a Bi-LSTM network to model the spatiotemporal dependencies of driving attention states. Achieving an attention state recognition accuracy of 93.2% in continuous driving scenarios.

(3) High-Accuracy, Low-Latency Driver Attention State Recognition

Through lightweight designs of the YOLO11n Pose and Bi-LSTM models, the RT-DASR response time is constrained within 21.5 ms, meeting the millisecond-level early warning requirements for driving safety. The proposed solution combines non-intrusiveness and low-cost characteristics, providing a viable technical pathway for the large-scale in-vehicle deployment of mining-truck driver attention state recognition systems.

## 2. Method

This section details the YOLO11n Pose detector for facial landmarks localization, binocular vision-based feature point distance measurement for spatial coordinate calculation, binocular vision-driven driver head pose angle estimation, driver gaze region partitioning for semantic area identification, and the architecture of the multi-source information fusion Bi-LSTM model for temporal feature extraction and driver attention state classification.

### 2.1. Introduction to the Real-Time Driver Attention State Recognition Component

**Figure 1:**
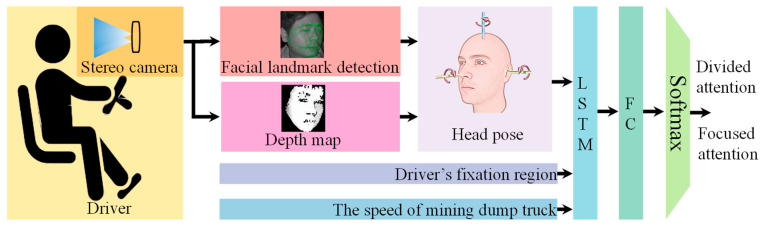
The framework of RT-DASR method.

The proposed driver attention state detection method is illustrated in Figure 1. This approach employs a binocular vision camera to capture facial images of the driver. The YOLO11n deep learning algorithm detects the facial region and locates 17 facial landmarks. Corner points less affected by facial expressions (e.g., eye corners, nostrils, outer eyebrow points) are selected as candidate points. Using the stereo image pair, a disparity map is computed to derive the 3D coordinates $\left(x_{i},y_{i},z_{i}\right),(i=1\sim n)$ of each candidate point in the world coordinate system, where *n* represents the number of candidate points. For each video frame, a rotation matrix is calculated based on the world coordinates of these candidate points. This matrix is subsequently decomposed to obtain the three Euler angles (yaw, pitch, roll) representing head orientation. These 3D head pose angles (yaw, pitch, roll), along with real-time vehicle speed and the driver’s gaze region, serve as input to the MSTBi-LSTM module. The Bi-LSTM network extracts temporal features from this multi-source data sequence. Finally, the output of the Bi-LSTM is fed into Fully Connected layers (FC) followed by a Softmax layer for classification, determining whether the driver’s attention state is divided or focused.

### 2.2. Introduction to the YOLO11n Pose Detector

**Figure 2:**
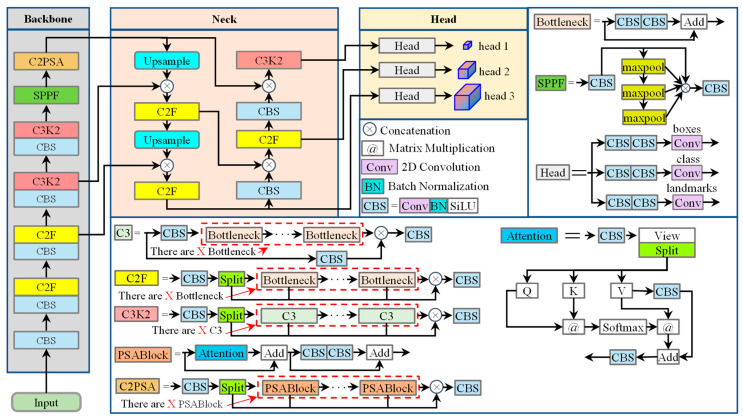
Structure of the YOLO11n Pose model.

In order to be able to perform fast facial landmark detection in embedded systems, we chose the Pose model in YOLO11n for face detection and facial landmark detection, and the structure of the YOLO11n Pose model is shown in Figure 2.

The YOLO11n Pose model primarily consists of three components: the Backbone, Neck, and Head, which are constructed using fundamental modules including CBS (Convolution BatchNorm SiLU), C3, C2F, C3K2, SPPF (Spatial Pyramid Pooling Fast), Bottleneck layers, and the Decoupled Head. The CBR module of Backbone performs feature map downsampling through a 3 × 3 convolution layer with stride 2, followed by Batch Normalization (BN) and a SiLU activation function. In the C3 module, the input feature map is first split into two parts: one part directly passes to a subsequent concatenation layer, while the other undergoes deep feature extraction via a Bottleneck module. The outputs from both paths are then concatenated along the channel dimension and compressed to the target channel number by a second CBS layer. This architecture enables efficient feature extraction and processing for pose estimation tasks.

The C2F module, designed based on the Cross Stage Partial (CSP) architecture, integrates two 1 × 1 CBS layers with stride 1 and multiple bottleneck layers. These bottleneck layers enhance gradient flow through residual connections, with each module containing two 3 × 3 convolution layers (stride 1) for high-level feature extraction. In processing, the input feature map first passes through the initial CBS layer, expanding the channel dimension to twice the input size. This output is then split into two branches: one directly routed to a subsequent concatenation layer, while the other undergoes deep feature extraction through multiple bottleneck modules. Finally, all bottleneck outputs are concatenated with the direct-pass features along the channel dimension, and the aggregated result is compressed to the target channel number by the second CBS layer.

The C3K2 module processes input feature maps by first propagating them through an initial CBS layer; the output is then bifurcated into two parallel paths: one branch directly feeds into a subsequent concatenation layer, while the other undergoes deep feature extraction via C3 modules. Ultimately, all processed outputs from the C3 modules are concatenated with the direct-pass features along the channel dimension, and the merged result is compressed to the target channel count through a subsequent CBS layer.

The C2PSA module implements a convolutional block incorporating attention mechanisms through the integration of a 1 × 1 convolutional layer and a series of PSABlock modules, thereby enhancing feature extraction and processing capabilities. Within this architecture, the input feature map initially traverses a primary CBS layer. The resultant output is subsequently partitioned into two branches: one directly propagates to a succeeding concatenation layer, while the other undergoes deep feature extraction via PSABlock modules. Ultimately, all processed outputs from the PSABlock modules are concatenated with the direct-pass features along the channel dimension, and the aggregated representation is compressed to the target channel count through a secondary CBS layer.

The PSABlock module enhances the model’s capacity to capture critical features within input data by integrating multi-head attention mechanisms with feed-forward neural network layers. The input feature map is first propagated to the attention module for executing multi-head attention mechanisms, enabling the model to focus on salient features across different spatial positions during data processing. The output from the attention mechanism is then added to the original input through residual summation, preserving source information while facilitating gradient flow. The resultant tensor subsequently traverses a CBS layer for further feature extraction and transformation. The final output tensor synthesizes representations processed by both the attention mechanism and the feed-forward network layer.

The Attention module implements a multi-head self-attention mechanism, enabling the model to focus on salient features at distinct spatial positions during input data processing. Initially, the input features are refined through a CBS layer for feature extraction, then projected into queries (Q), keys (K), and values (V) via View and Split operations. Subsequently, matrix multiplication is performed between the transposed K matrix and Q to generate the attention score matrix. Following this, the attention scores are scaled by a normalization factor to mitigate vanishing gradients. Softmax activation is then applied to normalize these scaled scores across the feature dimension. The normalized attention weights are used to compute a weighted sum over the values (V), yielding the attention output. Finally, this output is projected back to the original dimensionality through a CBS layer.

The SPPF module enhances the model’s receptive field through multi-scale feature fusion while reducing computational redundancy. Its architecture incorporates two CBS layers with stride = 1 and 1 × 1 kernels. The input features initially traverse the first CBS layer, then are partitioned into dual pathways: one directly propagates to a subsequent concatenation layer, while the other undergoes feature extraction through three successive 5 × 5 max-pooling layers arranged in cascade. Following this, the pooled features and CBS processed features undergo channel concatenation. The merged output is then compressed to the target channel dimensionality via the second CBS layer. The decoupled head comprises two distinct branches: a classification branch for category prediction and a regression branch for boundary box estimation. Each branch contains two CBR blocks with 3 × 3 kernels (stride = 1), followed by a 1 × 1 convolutional layer (stride = 1). The unified detection head employs a 2D convolution layer to simultaneously generate category probabilities, confidence scores, and bounding box coordinates.

Within the YOLO11n Pose architecture, input images first traverse a CBS layer for initial feature extraction in the Backbone. The features subsequently undergo iterative processing: initially passing sequentially through CBS and C2F modules (executed twice), then through CBS and C3K2 modules (also repeated twice). Finally, the feature maps proceed sequentially through SPPF and C2PSA modules for hierarchical feature extraction. Output feature maps from the second C2F, second C3K2, and C2PSA modules in the Backbone are designated as [C3, C4, C5] respectively. These Backbone outputs then enter the Neck module, where C5 is first upsampled via nearest-neighbor interpolation. The upsampled features undergo channel concatenation with C4, followed by feature refinement through a C2F module. This refined output is again upsampled and concatenated with C3, then processed through another C2F module to generate the P3 feature map. The P3 map traverses a CBS layer before concatenation with C4, with the fused features fed into C2F to yield P4. Similarly, P4 passes through CBS and concatenates with C5, then advances through a C3K2 module to produce P5. The resultant [P3, P4, P5] feature pyramids are finally distributed to three detection heads for simultaneous prediction generation.

### 2.3. Binocular Vision-Based Feature Point Distance Measurement

In binocular vision systems, the pinhole camera model is conventionally employed to describe the imaging process. Consider a camera with its optical center at point *O*. The relationship between the image plane and camera coordinate system is defined as follows: the origin of the camera coordinate system coincides with the optical center *O*, while the optical axis remains perpendicular to the image plane. The pixel coordinates (*x*, *y*) in the image plane are related to the 3D points (*X*, *Y*, *Z*) in the camera coordinate system as follows:

(1) $$x=\frac{fX}{Z},\;y=\frac{fY}{Z}$$

where *f* denotes the camera’s focal length, while *Z* represents the distance from the 3D point to the optical center *O* along the optical axis corresponding to depth information.

A binocular vision system typically comprises left and right cameras separated by a horizontal spatial interval known as the baseline distance *B*. The optical centers of the left and right cameras are denoted as *O_l_* and *Oᵣ*, respectively. For a 3D point *P* (*X*, *Y*, *Z*) in the scene, its projections on the image planes of the left and right cameras are *pₗ* (*xₗ*, *yₗ*) and *pᵣ* (*xᵣ*, *yᵣ*). Under ideal conditions—where both camera optical axes are strictly parallel and focal lengths are identical *f*—the similar triangle principle yields the fundamental stereo correspondence:

(2) $$x_{l}=\frac{fX}{Z},\;y_{l}=\frac{fY}{Z}$$

(3) $$x_{r}=\frac{f\left(X-B\right)}{Z},\;y_{r}=\frac{fY}{Z}$$

We derive the horizontal disparity *d* between corresponding image points.

(4) $$d=\frac{fB}{Z}$$

We establish the critical depth-disparity relationship as follows:

(5) $$Z=\frac{fB}{d}$$

In operational practice, binocular cameras capture left and right viewpoint images of the same scene. Stereo matching algorithms are then applied to generate a disparity map, which is converted to a depth map through this geometric principle. Candidate facial landmarks are identified on the depth map to locate registered points, enabling depth measurement from these features to the camera plane.

### 2.4. Depth-Compensated Head Pose Estimation

To enhance the accuracy of driver head-pose estimation, depth information was explicitly incorporated into the estimation framework. Driver head rotation exhibits three degrees of freedom in the coordinate system: pitch (rotation about the *x*-axis), in-plane roll (rotation about the *y*-axis), and yaw (rotation about the *z*-axis). For gaze direction analysis, visual transitions primarily arise from coupled yaw-pitch motions. Given depth measurements of facial landmarks obtained via binocular vision, driver pose estimation proceeds in three computational stages: First, world coordinates of the landmarks are computed using the camera’s intrinsic matrix **K** and extrinsic parameters [**R**|**t**]. Second, the rotation matrix **R** is derived from these feature coordinates with depth information through singular value decomposition. Finally, the three Euler angles (pitch, roll, yaw) are extracted from **R** via matrix decomposition. The camera intrinsic matrix **K**, rotation matrix **R**, and translation vector **t** are given by:

(6) $$K\;=\;\left[\begin{array}{ccc} f_{x} & 0 & c_{x}\\ 0 & f_{y} & c_{y}\\ 0 & 0 & 1 \end{array}\right]$$

(7) $$\left[R|t\right]=\left[\begin{array}{cccc} r_{11} & r_{12} & r_{13} & t_{x}\\ r_{21} & r_{22} & r_{23} & t_{y}\\ r_{31} & r_{32} & r_{33} & t_{z}\\ 0 & 0 & 0 & 1 \end{array}\right]$$

Given the image coordinates (*u*, *v*), their transformation into distortion-free normalized camera coordinates $\left(x_{c},\;y_{c}\right)$ yields the following rigid transformation between world and camera coordinate systems:

(8) A·X = b

(9) $$A=\left[\begin{array}{ccc} r_{11} & r_{12} & -x_{c}\\ r_{21} & r_{22} & -y_{c}\\ r_{31} & r_{32} & -1 \end{array}\right]$$

(10) $$X=\left[\begin{array}{c} X_{w}\\ Y_{w}\\ Z_{c} \end{array}\right]$$

(11) $$b=-\left[\begin{array}{c} t_{x}\\ t_{y}\\ t_{z} \end{array}\right]$$

The coordinates of the feature points in the world coordinate system can be obtained by solving the following system of equations.

(12) $$X={\;A}^{-1}\cdot b$$

Given a feature point with world coordinates $P_{1}\left(X_{1},Y_{1},Z_{1}\right)$ in the previous frame and $P_{2}\left(X_{2},Y_{2},Z_{2}\right)$ in the current frame, the rigidity constraint of 3D Euclidean transformation implies:

(13) $$P_{1}\;=\;R\cdot P_{2}$$

Consequently, the rotation matrix **R** can be determined, enabling subsequent derivation of the three attitude Euler angles. For the eight pre-selected feature points processed through this method, the computed Euler angles are averaged to obtain the driver’s final 3D rotational pose angles.

### 2.5. Driver Gaze Region Partition

**Figure 3:**
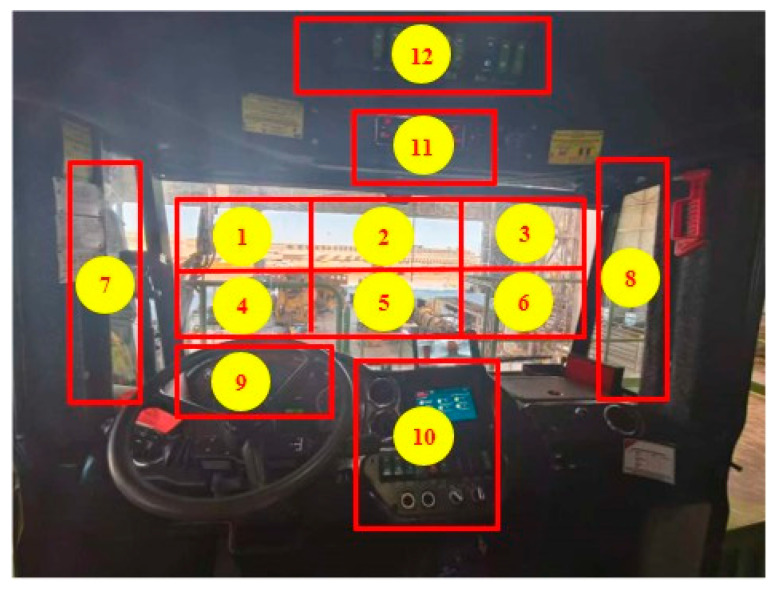
Schematic representation of driver gaze region distribution.

To transform driver gaze regions into semantically meaningful information for driving safety and thereby distinguish safe attention from distraction behavior, this study partitions gaze areas according to three principles: functional relevance (regions must correspond to driving task requirements, e.g., defining the forward roadway as a mandatory observation zone), spatial rationality (physical boundaries are delineated based on actual cabin layouts such as instrument clusters and common mobile device placement areas), and quantifiability (regions require explicit boundaries to support objective computation of distraction levels through duration and frequency metrics). This framework enables precise determination of whether attention is directed appropriately. Based on these principles, the driver’s viewpoint distribution within the mining truck cabin is divided into 12 discrete regions, allowing viewpoint locations to be represented numerically (1–12), the resulting cabin segmentation is illustrated in Figure 3.

### 2.6. Multi-Source Information Fusion Bidirectional Long Short-Term Memory

To integrate multi-sensor data and effectively extract temporal features for driver attention assessment, this study establishes MSTBi-LSTM capable of learning long-term dependencies. The proposed MSTBi-LSTM fuses sequential data pertaining to driver head pose, gaze regions, and vehicle speed, modeling attention states from these temporal patterns to determine driver attentiveness. Bi-LSTM, derived from LSTM, structurally differs through its bidirectional architecture comprising forward and reverse propagating recurrent networks, whereas standard LSTM employs unidirectional processing. Fundamentally, LSTM units consist of forget gates, input gates, memory cell states, and output gates. Specifically, the forget gate $f_{t}$ employs a sigmoid function to determine information discarded from the hidden layer, computed as:

(14) $$f_{t}=\sigma \left(W_{xf}x_{t}+W_{hf}h_{t-1}+b_{f}\right)$$

where $x_{t}$ denotes the input at time *t*, $h_{t-1}$ represents the hidden state at time t−1, $f_{t}$ indicates the output state of the forget gate at time *t*, *σ* signifies the sigmoid activation function, and $W_{f}$ and $b_{f}$ correspond to the weight matrix and bias vector of the forget gate, respectively. For inputs $x_{t}$ and $h_{t-1}$, the forget gate yields a value within [0, 1]; an output of 0 signifies complete discarding of the memory cell $c_{t-1}$ content, while an output of 1 indicates full retention. The input gate $i_{t}$ regulates information retention from the current input, computed as:

(15) $$i_{t}=\sigma \left(W_{xi}x_{t}+W_{hi}h_{t-1}+b_{i}\right)$$

where $x_{t}$ denotes the input at time *t*, $h_{t-1}$ represents the hidden state at time t−1, $i_{t}$ indicates the output state of the input gate at time *t*, with $W_{i}$ and $b_{i}$ being the corresponding weight matrix and bias vector for the input gate, respectively; the LSTM unit produces a candidate memory cell state vector ${\tilde{c}}_{t}$ via a tanh function, computed as:

(16) $${\tilde{c}}_{t}=tanh\left(W_{xc}x_{t}+W_{hc}h_{t-1}+b_{c}\right)$$

The LSTM updates the memory cell state from $c_{t-1}$ to $c_{t}$ through its forget gate and input gate, computed as:

(17) $$c_{t}=f_{t}\;⁎\;c_{t-1}+i_{t}\;⁎\;{\tilde{c}}_{t}$$

The output gate governs the proportion of the memory cell state $c_{t}$ that is propagated to the current hidden state $h_{t}$ of the LSTM, with its computation and the hidden state update formulated as:

(18) $$o_{t}=\sigma \left(W_{xo}x_{t}+W_{ho}h_{t-1}+b_{o}\right)$$

(19) $$h_{t}=o_{t}tanh\left(c_{t}\right)$$

where $o_{t}$ denotes the output state of the output gate at time *t*, with $W_{o}$ and $b_{o}$ being its corresponding weight matrix and bias vector, respectively.

**Figure 4:**
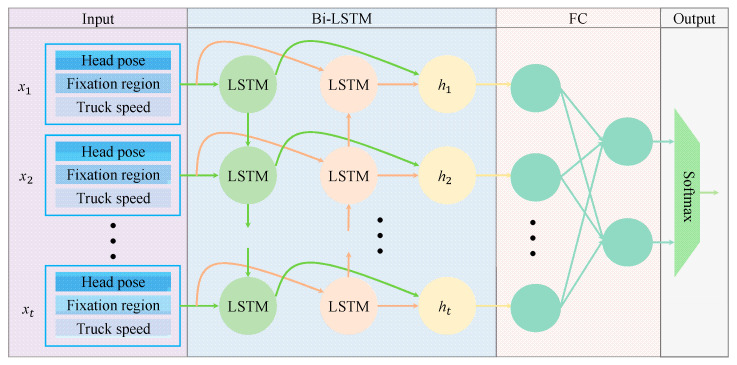
Structure of the MSTBi-LSTM model.

The architecture of the MSTBi-LSTM model is illustrated in Figure 4. The model accepts multiple sets of input sequences, each comprising: (1) the driver’s head pose, (2) the focal region of the driver’s gaze, and (3) the vehicle’s velocity. During Bi-LSTM processing, input sequences are fed into two separate LSTM networks in chronological and reverse-chronological order for feature extraction. The network then concatenates the output vectors from both LSTMs to form the final output at each time step. The forward propagation of the hidden layer output sequence in MSTBi-LSTM is expressed as:

(20) $${\vec{h}}_{t}=LSTM\left(x_{t},{\vec{h}}_{t-1}\right)$$

where ${\vec{h}}_{t}$ denotes the hidden state of the forward LSTM unit at time *t*, $x_{t}$ the input value at time *t*, and ${\vec{h}}_{t-1}$ the hidden state of the forward LSTM unit at time *t*−1.

The backward propagation of the hidden-layer output sequence in MSTBi-LSTM is formulated as:

(21) $${\overset{\leftarrow }{h}}_{t}=LSTM\left(x_{t},{\overset{\leftarrow }{h}}_{t-1}\right)$$

where ${\overset{\leftarrow }{h}}_{t}$ denotes the hidden state of the backward LSTM unit at time *t*, $x_{t}$ the input value at time *t*, and ${\overset{\leftarrow }{h}}_{t-1}$ the hidden state of the backward LSTM unit at time *t*−1.

As illustrated in Figure 4, the output of Bi-LSTM is fed into a FC layer, ultimately generating the driver’s attention state through a softmax classifier. The feature dimensions per time step in the proposed MSTBi-LSTM model comprise: (1) three degrees of freedom (head pose), (2) twelve gaze regions, and (3) one vehicle speed metric, resulting in an input dimension of 16. The MSTBi-LSTM configuration employs a sequence length of 300, 128 hidden units, and 1 layer.

## 3. Experiment, Results and Discussion

### 3.1. Experimental Environment Settings and Dataset

Model Training Environment: the experimental hardware comprised an Intel Xeon Silver 4210 processor and an NVIDIA RTX 3090 GPU. Software configurations included Ubuntu 22.04 LTS, PyTorch 2.3, CUDA 11.2, ONNX 1.8, and Python 3.12. Deployment Environment: the embedded terminal processor was deployed on an NVIDIA Jetson Orin platform running Ubuntu 20.04 OS, with JetPack 5.1.4 and TensorRT 8.5. To guarantee stable operation under low-illumination conditions, the stereo rig incorporates a near-infrared (NIR) LED illuminator whose dominant emission wavelength is fixed at 940 nm. The baseline of the system is precisely set to 43 mm, and the optics are equipped with lenses of 3.5 mm focal length.

The YOLO11n Pose model was trained on a dataset comprising 9798 images, partitioned into training, validation, and test sets at a 6:2:2 ratio. Training employed a learning rate of 0.01 with Stochastic Gradient Descent (SGD) optimization, mixed-precision acceleration, and mosaic data augmentation. The model underwent 300 epochs with early stopping triggered after 100 consecutive epochs of no improvement. Evaluation metrics included precision (ratio of true positives to predicted positives), recall (ratio of true positives to actual positives), AP_50–95_ (mean average precision across intersection over union thresholds 0.5–0.95 in 0.05 increments), parameter count, computational complexity (GFLOPs), inference time, and post-processing time where precision and recall serve as core detection metrics, AP_50-95_ reflects comprehensive localization performance.

For MSTBi-LSTM, training and testing data originated from driver videos segmented into 60 s clips (1500 frames at 25 fps). Training sampled every 5 frames, with features comprising normalized 3D Euler angles, gaze region $S_{k}$ (1~12), and vehicle speed $V_{k}$ per frame k. The dataset contained 5000 samples (3000 training, 1000 validation, 1000 test). Training configuration used batch size 128, 300 epochs, 0.001 learning rate, Binary Cross Entropy (BCE) loss, and Adam optimization, halted after 100 epochs without accuracy improvement. Primary evaluation metrics were accuracy and inference time.

The performance of BV-DHPE was evaluated using mean absolute error, root mean square error (RMSE), and standard deviation (SD).

### 3.2. Model Training and Testing for Face Detection and Facial Landmark Detection

To verify operational compliance of YOLO11n Pose, comparative experiments were conducted using YOLOv8n Pose, YOLO11n Pose, and YOLO12n Pose models for face detection and facial landmark detection. Quantitative results for face detection performance are presented in Table 1.

As shown in Table 1, YOLO11n Pose achieved a precision of 99.8%, surpassing YOLOv8n Pose (99.7%) by 0.1 percentage points, indicating the lowest false detection rate. YOLO12n Pose (99.6%) showed marginally lower precision than both predecessors. YOLO11n Pose attained a recall of 99.9%, outperforming YOLOv8n Pose (99.6%) and YOLO12n Pose (99.8%), demonstrating optimal control over missed detections.

Both YOLOv8n Pose and YOLO12n Pose achieved an AP_50-95_ of 89.2%, slightly below YOLO11n Pose’s 90.7%, confirming the latter’s superior average precision in object detection tasks. YOLO11n Pose and YOLO12n Pose exhibited parameter counts of 2.9 million and 2.8 million, respectively, significantly lower than YOLOv8n Pose’s 3.3 million. Additionally, YOLO11n Pose and YOLO12n Pose has 7.4 GFLOPs computational cost, indicating lower computational complexity compared to YOLOv8n Pose’s 9.2 GFLOPs.

In summary, YOLO11n Pose demonstrates exceptional performance in precision, recall, and average precision while outperforming YOLOv8n Pose in parameter efficiency and computational complexity despite having 0.1 million more parameters than YOLO12n Pose. These characteristics confirm YOLO11n Pose’s suitability for deployment in computationally constrained environments without compromising high accuracy.

To demonstrate YOLO11n Pose’s superior capability in facial landmark detection, quantitative experimental results for YOLOv8n Pose, YOLO11n Pose, and YOLO12n Pose are presented in Table 2.

As shown in Table 2, YOLO11n Pose demonstrates the highest recall (99.9%), while YOLOv8n Pose and YOLO12n Pose achieve 99.6% and 99.7% recall respectively. Regarding precision, both YOLO11n Pose and YOLOv8n Pose attain 99.7%, with YOLO12n Pose slightly lower at 99.5%. In this study, YOLO11n Pose achieves the highest AP_50-95_ of 94.5%, indicating superior average precision across intersection over union thresholds, whereas YOLOv8n Pose and YOLO12n Pose score 93.0% and 93.1% respectively. Inference time and post-processing time serve as critical metrics for computational efficiency evaluation: YOLO11n Pose delivers optimal inference latency at 1.1 ms, marginally outperforming YOLOv8n Pose (1.1 ms) and YOLO12n Pose (1.7 ms). For post-processing time, YOLO11n Pose similarly excels at 1.0 ms compared to YOLOv8n Pose’s 1.2 ms and YOLO12n Pose’s 1.3 ms. Cumulatively, YOLO11n Pose surpasses counterparts in recall, inference efficiency, and post-processing speed, validating its efficacy and accuracy for facial landmark detection tasks; consequently, this research adopts YOLO11n Pose for both face detection and facial landmark localization.

### 3.3. Comparative Experiments on Driver Head Posture Estimation

**Figure 5:**
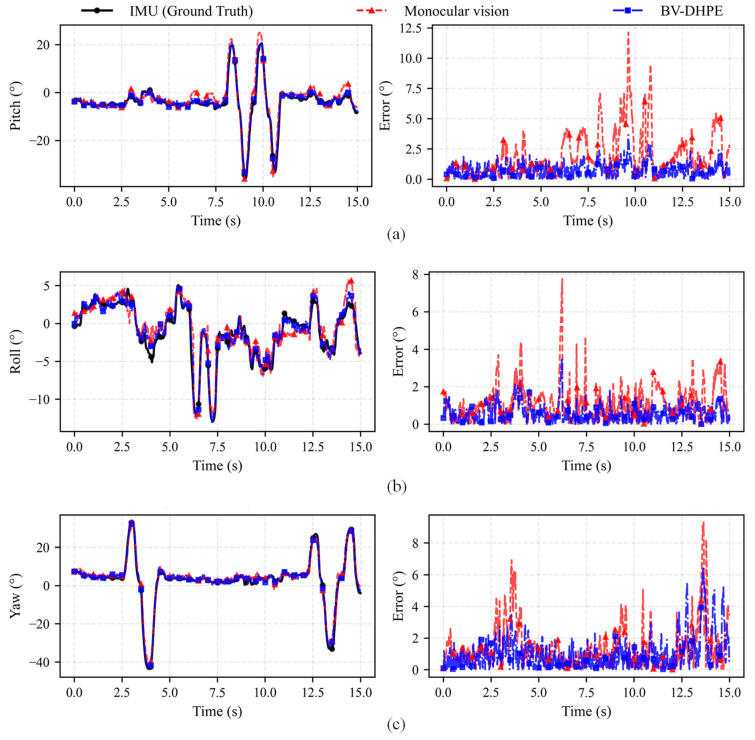
Head Pose estimates derived from distinct methodologies and their quantitative error from ground truth. (**a**) experimental results of pitch angle; (**b**) experimental results of roll angle; (**c**) experimental results of yaw angle.

To demonstrate the superior accuracy of the proposed head pose estimation method over monocular approaches, a comparative experiment was conducted. The IM600 sensor (Chenyi Electronic Technology Factory, Zhongshan City, China) captured ground-truth driver head poses, while pose estimations were simultaneously generated using both conventional monocular vision methods and the proposed BV-DHPE. Figure 5 presents the Euler angles of head pose estimated through distinct methodologies, along with their quantitative error from ground truth.

As illustrated in Figure 5, experimental validation across pitch, roll, and yaw axes reveals that both monocular vision and the proposed BV-DHPE framework deliver accurate Euler-angle estimates under quasi-static conditions when referenced against IMU-derived ground truth. When the head undergoes rapid angular excursions, however, both modalities exhibit error amplification; yet the magnitude and sensitivity differ markedly between approaches. In the pitch axis, monocular vision registers errors up to 12.1°, whereas BV-DHPE confines the peak error to 3.6°. A similar trend is observed for roll and yaw: monocular estimates reach 9.3° at extreme angles, while BV-DHPE maintains errors below 6.5°. This consistent disparity is attributed to the degradation of facial-landmark reliability at large angles, which disproportionately degrades monocular precision. By integrating depth-constrained refinement, BV-DHPE attenuates these effects, sustaining sub-degree accuracy even under high-amplitude motion and corroborating the critical contribution of geometric constraints to robust pose estimation.

**Figure 6:**
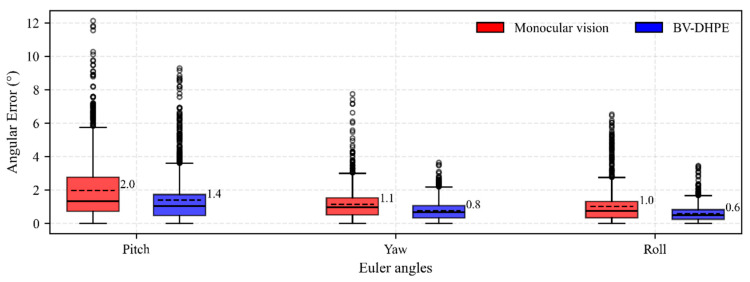
Box-and-whisker plots comparing head pose estimation methods.

Figure 6 presents box-and-whisker plots comparing the angular errors (in degrees) for pitch, yaw, and roll Euler angles estimated using monocular vision and the proposed BV-DHPE method. BV-DHPE consistently demonstrates superior performance across all axes, yielding lower median errors, reduced extreme deviations, and tighter error distributions compared to the monocular baseline. Specifically, for pitch estimation, the monocular approach yields MAE = 2.0°, RMSE = 2.7°, median error = 1.3°, maximum error = 12.1°, and SD = 1.8°. BV-DHPE significantly reduces these metrics to 0.8°, 0.9°, 0.7°, 3.6°, and 0.6°, respectively, achieving a 60.0% improvement in MAE. In the yaw direction, monocular vision registers MAE = 1.4°, RMSE = 2.0°, median = 1.0°, maximum = 9.3°, and SD = 1.4°; BV-DHPE lowers these to 1.0°, 1.4°, 0.7°, 6.5°, and 1.0°, corresponding to a 28.6% MAE reduction. Similarly, for roll, monocular results (MAE = 1.1°, RMSE = 1.5°, median = 1.0°, maximum = 7.7°, SD = 0.9°) are improved by BV-DHPE to 0.6°, 0.8°, 0.5°, 3.5°, and 0.5°, representing a 45.5% MAE reduction. BV-DHPE achieved 44.7% reduction in head pose MAE compared to monocular vision methods. These results substantiate that integrating binocular depth cues effectively mitigates scale ambiguity inherent in monocular systems, substantially enhancing both the accuracy and robustness of head-pose estimation.

### 3.4. Model Training and Testing for Multi-Source Temporal Bidirectional Long Short-Term Memory

To demonstrate that MSTBi-LSTM’s fusion of multi-sensor data enhances predictive performance, ablation experiments were conducted, with results presented in Table 3. This table delineates the impact of different data sources combinations on MSTBi-LSTM’s performance, specifically evaluating three features: head pose, fixation region, and truck speed. The study aims to assess how these combinations affect the model’s accuracy and inference time.

When solely utilizing head pose as input, MSTBi-LSTM achieved 85.2% accuracy. This indicates that head pose constitutes an effective feature capable of providing sufficient information for classification tasks. However, reliance on a single feature constrains the model’s capacity to capture complex patterns, thereby limiting overall performance.

Upon introducing the fixation region feature, accuracy increased to 89.7%. This improvement confirms the complementary relationship between fixation region and head pose features, enriching informational input and enhancing classification performance. Notably, inference time remained unchanged despite the added feature, demonstrating the model’s computational efficiency.

With the further inclusion of truck speed, accuracy rose to 93.2%. This result underscores the criticality of feature complementarity, as the dynamic temporal characteristics of vehicle speed provide behavioral cues that strengthen predictive capability. Crucially, inference time persisted at 0.1 ms even with three features, attesting to MSTBi-LSTM’s efficiency and stability in multi-feature processing.

The MSTBi-LSTM model was trained using five-fold cross-validation on the combined training and validation datasets. As shown in Table 3, the accuracy achieved by MSTBi-LSTM through five-fold cross-validation is 0.3% higher than that obtained using only the validation set. The similarity in the results between the two approaches suggests that the training process yields a stable outcome.

Table 3 reveals a progressive accuracy enhancement with feature augmentation while inference time remains stable. This demonstrates that strategic feature selection and fusion can substantially boost model performance without incurring significant computational overhead. The most pronounced accuracy gain occurred with truck speed integration, likely attributable to its delivery of critical dynamic driving behavior signals that empower MSTBi-LSTM to better interpret and predict driving states.

### 3.5. Deployment Experiment of BV-DHPE and MSTBi LSTM

To demonstrate sustained high performance of YOLO11n Pose and MSTBi-LSTM when deployed on Jetson Orin, we conducted deployment experiments. The trained YOLO11n Pose model was exported to TensorRT format and executed with FP16 precision on Jetson Orin Nano. Experimental results are presented in Table 4.

As shown in Table 4, metrics including precision, recall, and AP_50-95_ decreased when using float16 precision compared to float32 on NVIDIA Jetson Orin. This degradation primarily stems from reduced numerical representation accuracy in float16, causing partial loss of fine-grained details that impairs detection accuracy. Due to the substantially lower computational power of NVIDIA Jetson Orin versus NVIDIA RTX 3090, inference latency increased significantly after deployment on NVIDIA Jetson Orin. Nevertheless, the model maintained real-time inference capability. Therefore, our approach employs float16 precision for model deployment.

The trained MSTBi-LSTM model was exported to TensorRT format and subsequently deployed on Jetson Orin for inference testing. Experimental results are presented in Table 5.

As shown in Table 5, when using float16 precision, the MSTBi-LSTM model trained with five-fold cross-validation experiences a 0.3% drop in accuracy and a 2-millisecond increase in inference latency. This performance trade-off is deemed acceptable; hence, our deployment framework employs float16 precision for MSTBi-LSTM inference.

### 3.6. In-Vehicle Testing of Driver Attention State Recognition Method

To comprehensively evaluate the performance of the RT-DASR method in real vehicular environments, this study employs four key metrics: Accuracy, TPR (True Positive Rate, recall), FPR (False Positive Rate, false detection rate), and detection latency. These metrics collectively assess RT-DASR’s discriminative capability and real-time performance from multiple perspectives.

The True Positive Rate (TPR) represents the proportion of actual distraction instances correctly identified by RT-DASR. This metric directly quantifies RT-DASR’s missed detection risk, indicating its ability to recognize genuine distraction events. Higher TPR values signify greater detection accuracy and lower missed detection risks. In practical applications, high TPR is critical for driving safety, effectively reducing potential accident risks caused by oversight.

The False Positive Rate (FPR) denotes the proportion of normal driving instances erroneously classified as distraction states by RT-DASR. This metric reflects the system’s false alarm risk, characterizing its tendency to misidentify normal driving behavior as distraction. Lower FPR values indicate higher recognition accuracy during normal driving and reduced false alarms. Maintaining low FPR is essential for optimizing user experience and minimizing unnecessary interventions.

Detection latency refers to the processing time required for RT-DASR to output attention state determinations, measured in milliseconds per frame. This metric directly determines system real-time performance, with timing benchmarks obtained from Jetson Orin platform measurements.

**Figure 7:**
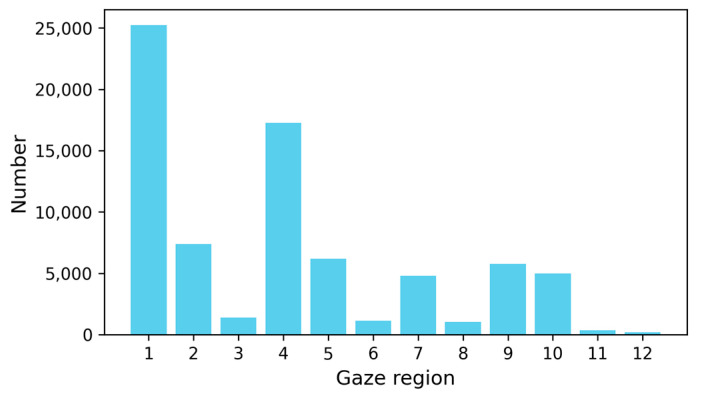
Distribution of driver gaze regions.

Experimental data comprises 1440 h of real-world operation records from 40 mining truck drivers, with video samples covering daylight (53%) and nighttime (47%) conditions. The collection of these data has been approved by 40 mining-truck drivers. From this dataset, 600 distracted-driving clips and 3000 non-distracted clips were annotated, each spanning 60 s. The distribution of the drivers’ gaze regions in the dataset is illustrated in Figure 7, which intuitively reflects the attention distribution of drivers when operating mining trucks. The gaze region with the highest frequency of drivers’ gaze is region 1, followed by region 4. This indicates that mining-truck drivers spend the majority of their time focusing on the road ahead. Gaze regions 2, 3, 5, and 6 represent the drivers’ scanning motions of the road conditions in front of the vehicle. Gaze regions 7 and 8 typically correspond to the drivers’ actions of observing vehicles behind. Gaze regions 9 and 10 usually represent the drivers’ observation of the dashboard. The relatively low frequency of gaze in regions 11 and 12 suggests that the information provided by these regions is less frequently required by the drivers.

A comparative experiment on driver attention detection was conducted using a test dataset comprising data from 40 mining-truck drivers, pitting the conventional monocular approach against the RT-DASR method. The detailed results of the experiment are presented in Table 6.

The monocular method demonstrates relatively lower performance in accuracy and TPR metrics at 80.1% and 80.4% respectively. This indicates limited accuracy and recall in identifying distraction levels. Furthermore, its higher FPR (19.8%) implies excessive false alarms during recognition, compromising practical reliability. A detection latency of 18.2 ms suggests satisfactory real-time performance.

RT-DASR exhibits significant advantages across all metrics. Achieving 90.4% accuracy and 90.7% TPR, the method demonstrates superior recognition accuracy and recall, indicating enhanced state discrimination capability through binocular depth information. The lower FPR (8.8%) further validates its effectiveness in reducing false alarms. The F1 score of RT-DASR is 12% higher than that of the monocular approach, indicating that RT-DASR maintains good performance even when the test data categories are imbalanced. Although the detection latency measures 21.5 ms, marginally higher than the monocular approach, this delay remains acceptable in practical applications, with performance benefits potentially offsetting the limitation.

**Figure 8:**
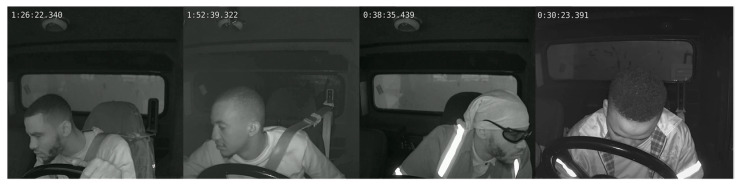
Failure case visualization of attention state recognition errors in RT-DASR.

Through visual analytics, we identified failure cases in RT-DASR recognition, with representative examples illustrated in Figure 8. As illustrated in Figure 8, false negatives in the proposed method primarily resulted from feature-point matching failures during extreme head rotations.

In summary, the proposed method achieves higher accuracy and reliability in distraction-level recognition tasks. Despite marginally increased detection latency, substantial improvements in accuracy, TPR, and FPR collectively demonstrate enhanced operational value for real-world deployment.

## 4. Conclusions

This study systematically evaluates RT-DASR for driver state recognition, integrating YOLO11n Pose facial landmark detection with MSTBi-LSTM temporal modeling. Key findings demonstrate that YOLO11n Pose achieves optimal balance between precision and computational efficiency (1.1 ms inference latency), outperforming comparable architectures in facial feature extraction. The progressive feature augmentation strategy reveals critical insights: fixation regions improve accuracy by 4.5%, while truck speed integration delivers a further 3.5% gain, collectively achieving 93.2% classification accuracy without compromising the 0.1 ms inference speed of MSTBi-LSTM.

The proposed BV-DHPE method demonstrably enhances head-pose estimation accuracy and robustness compared to monocular vision. Quantitative evaluation revealed significant reductions in angular error metrics across all Euler angles (pitch, yaw, roll) when using BV-DHPE. Specifically, MAE reduction of 44.7% was achieved, alongside markedly tighter error distributions and lower extreme deviations. These results demonstrate that binocular depth compensation effectively reduces errors in monocular head pose estimation caused by depth deficiency, enabling more accurate and robust pose estimation under dynamic conditions.

The proposed RT-DASR system exhibits significant advantages over monocular approaches, with 10.3% higher accuracy (90.4%) and 11.0% lower FPR (8.8%), validating the effectiveness of binocular depth information for distraction recognition. Despite a 3.3 ms latency increase compared to monocular systems, the 21.5 ms processing time remains practical for real-world deployment. When deployed on edge devices, Float16 inference maintains functional feasibility with accuracy degradation within 0.3%, overcoming computational constraints while preserving real-time performance.

This study has its limitations. Compared with conventional vehicles, mining trucks are substantially larger, demand a broader visual field from the operator, and require simultaneous monitoring of multiple control panels and displays. These characteristics increase the prevalence of behaviors that are classified as non-safety-critical distractions. Consequently, distraction-detection methodologies developed for mining trucks are not readily transferable to standard passenger vehicles. Furthermore, the dataset employed herein diverges markedly from those utilized in prior work, precluding the establishment of a unified evaluation benchmark and rendering direct comparison with state-of-the-art distraction-detection approaches impractical.

Error analysis indicates extreme head rotations as the primary failure mode, suggesting need for robust feature-point matching. This study advances driver monitoring systems by establishing a robust benchmark for real-time attention recognition. Subsequent research will focus on wide-angle head rotation scenarios, employing multi-camera configurations to construct multi-view stereo vision. This approach will achieve comprehensive head rotation coverage, thereby preventing pose estimation failures caused by missing facial landmarks.

## Figures and Tables

**Table 1:** Comparative face detection results across models.

Model	Precision (%)	Recall (%)	AP_50-95_ (%)	Parameters (M)	GFLOPs
YOLOv8n Pose	99.7	99.6	89.2	3.3	9.2
YOLO11n Pose	99.8	99.9	90.7	2.9	7.4
YOLO12n Pose	99.6	99.8	89.2	2.8	7.4

**Table 2:** Comparative facial landmark detection results across models.

Model	Precision (%)	Recall (%)	AP_50-95_ (%)	Inference (ms)	Postprocess (ms)
YOLOv8n Pose	99.7	99.6	93.0	1.1	1.2
YOLO11n Pose	99.7	99.9	94.5	1.1	1.0
YOLO12n Pose	99.5	99.7	93.1	1.7	1.2

**Table 3:** Impact of distinct input data sources on MSTBi-LSTM performance.

Head Pose	Fixation Region	Truck Speed	Five-Fold Cross-Validation	Accuracy (%)	Inference (ms)
√				85.2	0.1
√	√			89.7	0.1
√	√	√		93.2	0.1
√	√	√	√	93.5	0.1

**Table 4:** Deployment experiment results of BV-DHPE.

Model	Precision (%)	Recall (%)	AP_50-95_ (%)	Inference (ms)
YOLO11n Pose	99.3	99.5	92.2	16.8

**Table 5:** Deployment experiment results of MSTBi-LSTM.

Model	Five-Fold Cross-Validation	Accuracy (%)	Inference (ms)
MSTBi-LSTM		93.0	2.1
MSTBi-LSTM	√	93.2	2.1

**Table 6:** Real vehicle experimental results of monocular method and RT-DASR.

Method	Accuracy (%)	F1 Score	TPR (%)	FPR (%)	Inference (ms)
monocular	80.1	80.3	80.4	19.8	18.2
RT-DASR	90.4	92.3	90.7	8.8	21.5

## Data Availability

The data presented in this study are available on request from the corresponding author.
